# Different routes the same destination: a comparative study of antitrust regulation for pharmaceutical industry in the United States and China

**DOI:** 10.3389/fphar.2025.1557876

**Published:** 2025-04-17

**Authors:** Jie Weng, Nailiang Liu

**Affiliations:** Center for Competition Law and Policy, School of Law, Chongqing University, Chongqing, China

**Keywords:** pharmaceutical industry, anti-monopoly regulation, antitrust regulation, United States of America, China, comparative study

## Abstract

As a commercial trade with a public nature, the pharmaceutical industry is related to the interests of many consumers. It is important for many countries to carry out antitrust regulations in this industry, but there are differences in regulatory paths and specific practices (terms such as “anti-monopoly” and “antitrust” are used interchangeably, this study uses “antitrust” to maintain consistency in terminology). A key characteristic of the United States system is that courts play a leading role in interpreting and applying abstract antitrust laws, whereas Chinese administrative enforcement agencies directly apply specifically identified antitrust provisions for uniform regulation. Despite differences, pharmaceutical antitrust regulations in both countries ultimately aim to protect consumer welfare. This study examines the timelines of pharmaceutical antitrust regulation, the fields of regulation, the types of monopoly behavior and behavioral performances in two countries, and further analyses the differences in the regulatory paths and means, as well as the similarities and differences in the effects of regulation, in comparison with the current state of pharmaceutical antitrust regulation in the United States and China. The analysis serves as a valuable reference for the future development of pharmaceutical antitrust regulation in China.

## 1 Introduction

According to public data from the World Bank (World Health Organization, 2000), the United States and China have shown an overall upward trend in health expenditures since 2000. The most recent data for 2021 shows that the U.S. spends 17.36 percent of its gross domestic product (GDP, gross domestic product) on health, more than four percentage points higher than the average for high-income countries. Chinese health spending is also gradually reaching the level of middle-income countries, attaining 5.38 percent of GDP by 2021.

The United States stands out as a country with the world’s largest consumer of pharmaceutical products (Gary, 2019) and is considered highly advanced in antitrust enforcement. As early as the mid-1970 s, the Goldfarb case confirmed that the healthcare sector, which is characterized as a “learned profession”, would also be subject to the jurisdiction of the Sherman Act, thus initiating the U.S. government’s antitrust scrutiny of potential antitrust violations in the healthcare sector, including the pharmaceutical industry (Federal Trade Commission, 2024). The Eli Lilly/PCS case was the first filed by the Federal Trade Commission (FTC, Federal Trade Commission) against the pharmaceutical industry. It is worth noting that when antitrust laws entered the pharmaceutical industry, the U.S. pharmaceutical industry had developed into a mature market with both the dividends of the times and policy incentives (Wang et al., 2018).

On the other hand, China has been the second-largest pharmaceutical market in the world for more than a decade (IQVIA, 2024). Since implementing the Antitrust Law in 2008, China initiated a series of antitrust enforcement programs targeting the pharmaceutical industry. Over the past 16 years (2008–2024), law enforcement efforts in this sector have steadily intensified, driven by the progressive refinement of regulations and policies. Key milestones include the introduction of the Fair Competition Review Mechanism in 2016 and the launch of specialized antitrust enforcement campaigns in people’s livelihood sectors in 2023, including the pharmaceutical industry. Despite the relatively short history of law enforcement, statistics show that of the 439 public cases of antitrust administrative enforcement in China in the past 16 years, the pharmaceutical industry accounts for 10%. It has become the highest incidence of antitrust administrative regulation cases (Liu and Chen, 2021).

The U.S.’s antitrust regulation of the pharmaceutical industry is more than 30 years earlier than China, which has more practical experience. Although the cultural backgrounds, legal systems and levels of industry development are different, the cooperation mechanism and common goals of antitrust regulation between the two countries have provided the possibility for mutual learning. This study is dedicated to analyzing the different paths of pharmaceutical antitrust regulation through a comparative analysis of administrative enforcement and judicial litigation public cases between the United States and China. Moreover, our focus extends to examining policy orientation and value convergence in the two economies, aiming to provide some actionable recommendations for antitrust regulation in China.

## 2 Methods

### 2.1 Cases retrieval

This study first collected publicly available pharmaceutical antitrust cases from China and the United States. Specifically, cases from 2008 to 2024 were retrieved from the official websites of Chinese antitrust enforcement agencies: the National Development and Reform Commission, the Ministry of Commerce, and the State Administration for Market Regulation (formerly the State Administration for Industry and Commerce) - as well as the Supreme People’s Court of China. For the United States, pharmaceutical antitrust cases spanning from the 1985 Eli Lilly/PCS case (a landmark antitrust case in the pharmaceutical industry) to 2024 were sourced from the public databases of U.S.’s antitrust agencies: the Federal Trade Commission, the Department of Justice (DOJ, the Department of Justice) and the U.S. Supreme Court. Additional case details were supplemented using legal databases such as Westlaw and LexisNexis. Furthermore, this study conducted searches using policy terminology commonly employed in competition enforcement documents and reports from China and the United States. Keywords such as “pharmacy antitrust,” “pharmacy anti-monopoly,” “anticompetitive mergers,” “医药反垄断 (pharmaceutical antitrust),” and “原料药垄断 (active pharmaceutical ingredient monopoly)” were applied, supplemented by Boolean operators (AND/OR) to expand the search scope. The objective was to compile as comprehensively as possible all publicly available cases related to antitrust reviews in the pharmaceutical industry.

### 2.2 Screening criteria

Cases were retrieved from the start of antitrust enforcement, focusing on drug research, development, manufacturing, and distribution to ensure data completeness and relevance. Meanwhile, duplicate cases and those lacking full disclosure of facts and legal basis were excluded. Ultimately, 61 Chinese cases and 140 U.S. cases (including pending cases) were included, covering all categories of administrative law enforcement, judicial rulings, and merger reviews.

### 2.3 In-depth analysis

A thorough reading of the selected case texts was undertaken. This study performs frequency statistics and proportion calculations on the filing time, the fields of regulation, and the types and behaviors of pharmaceutical anti-monopoly violations in the two countries. It creates comparative charts and conducts a comparative analysis of their respective antitrust enforcement approaches.

### 2.4 Quality control

To ensure reliability and validity of the findings, a double data entry method was applied during data collection and analysis. It also conducted a multi-source data verification by retrieving selected cases from U.S. - China competition enforcement documents and report texts to minimize biases in collection and interpretation.

### 2.5 Literature synthesis

By citing legal frameworks, integrating academic theories, and applying data verification methods, this study transforms multi-type literature into a foundation for comparative research.

### 2.6 Reflection and critical evaluation

Based on the comprehensive research, the study used institutional economics to examine how the antitrust regimes influence pharmaceutical market behavior and structure. Based on this primary theoretical framework, this study reflected on the effectiveness of pharmaceutical antitrust in the United States and China, providing suggestions for improvements to Chinese pharmaceutical antitrust regulation.

## 3 Comparison of cases in the antitrust regulation for pharmaceutical industry in the United States and China

### 3.1 Regulatory timeline

**FIGURE 1:**
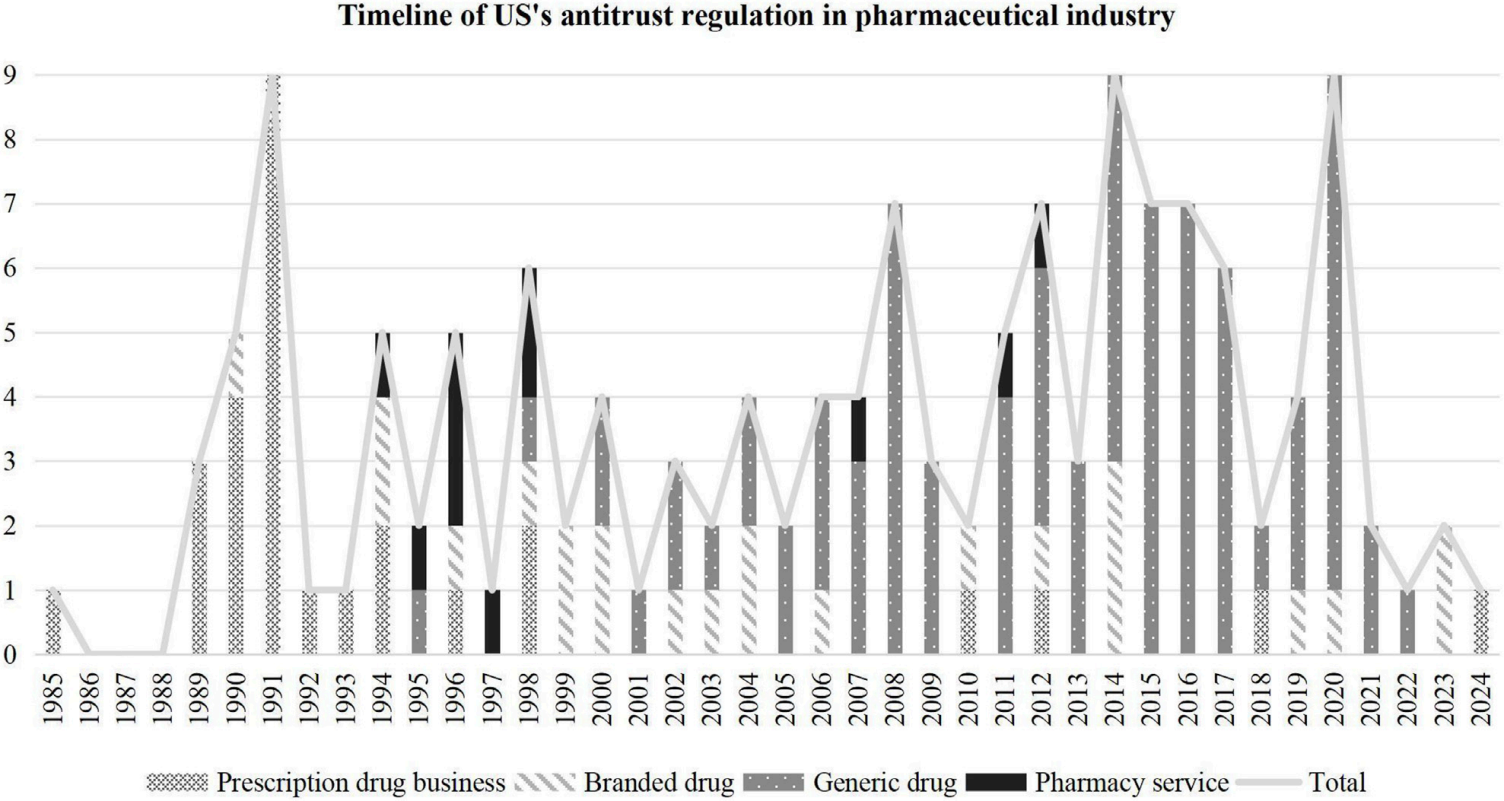
Timeline of US’s antitrust regulation in the pharmaceutical industry (Based on filing time).

This study divides the timeline of U.S.’s pharmaceutical antitrust regulation, as reflected in Figure 1 into three parts:

Part I: Regulatory focus on prescription drug services before 2000. In the 1990 s, managed care firms developed in the United States. This kind of company can negotiate prices directly with healthcare providers to reduce patient expenditures. During this period, managed care firms concentrated their cost control efforts on the prescription drug business. However, this entailed reduced reimbursements to pharmacies and decreased in dispensing fees (Janet, 1996). From 1989 to 1998, anti-competitive plans to resist negotiated programs that aim at reducing the cost of prescription drugs were investigated by the FTC, notably in the case of the Chain Pharmacy Association of New York State. Between 1989 and 1992, the FTC initiated 17 complaints alleging that numerous retail pharmacy chains, their trade associations, independent pharmacy trade associations, and two individuals illegally conspired to boycott New York State’s Employee Prescription Plan (Janet, 1996).

Part II: The shift in regulatory focus to the generic market after 2000. In 2000, the FTC formally announced the study of generic drug competition in response to anti-competitive behaviors by pharmaceutical companies abusing the Hatch-Waxman Act (Federal Trade Commission, 2000). Specifically, the FTC initiated the first review of a patent settlement agreement against three pharmaceutical companies in 2000. Since then, the FTC began to regulate anti-competitive behaviors of generic and branded companies that conspired to “park” the 180-day exclusivity for first-filing generic and delay its entry into the marketplace (Federal Trade Commission, 2002). In addition, since 2002, antitrust reviews have also been conducted against abusive patent-infringement litigation by branded firms using procedures such as the 30-month stay provisions to sue generic manufacturers. Furthermore, the FTC regulated other violations of federal antitrust policy by generic drug companies. Since 2016, the DOJ also joined the ongoing federal antitrust investigation into price fixing, bid rigging, and other anti-competitive behavior in the generic pharmaceutical industry (US Department of Justice, 2016).

Part III: Continuing regulation of anti-competitive mergers under the Hart-Scott-Rodino Antitrust Improvements Act of 1976. This act, introduced as an essential amendment to the Clayton Act, requires companies engaging in particular mergers and acquisitions to notify the two antitrust enforcement agencies in advance for an anti-competitive impact review. Since the Eli Lilly/PCS case in 1985, the FTC and the DOJ have been continuously supervising large mergers and acquisitions activities in pharmaceutical industry and have been influenced by the shift of regulatory emphasis. Before 2000, relevant cases were mainly the mergers of large pharmacies. After 2000, the mergers and acquisitions between branded companies and generic companies became the focus of review. It is worth noting that mergers and acquisitions between innovative branded companies have also been under regulation, typically the acquisitions of companies developing optimized products to challenge the present patent drugs.

Table 1 reflects changes in the regulatory focus and significant cases of United States’s pharmaceutical antitrust.

**TABLE 1:** Changes in the regulatory focus and significant cases of US’s pharmaceutical antitrust.

Regulatory focus
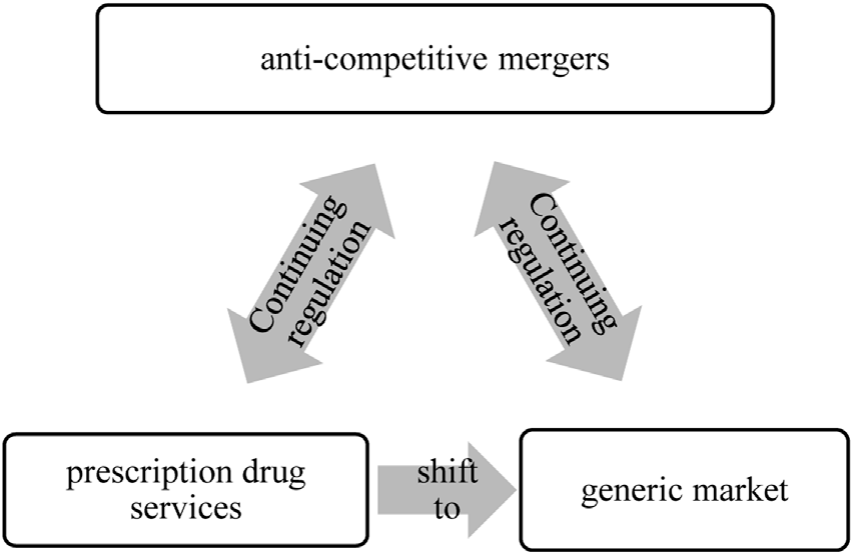

Significant cases		
Year	Cases	Description
1975	In the case of Goldfarb	The healthcare industry will also be subject to the Sherman Act
1985	In the matter of Eli Lilly/PCS	The first antitrust regulation in the pharmaceutical industry by the FTC
1990	In the case of The Procter & Gamble Company	The first antitrust regulation in the pharmaceutical industry by the DOJ
1985–2000	In the matter of Genovese Drug Stores, Inc.	30 cases of regulating the monopoly of pharmacy on prescription drug business and service 20 cases of regulating anti-competitive mergers and acquisitions
	In the matter of Carl’s Drug Co., Inc.	
	In the matter of Brooks Drug, Inc.	
	……	
2000	In the matter of Hoechst Marion Roussel, Inc./Carderm Capital L.P.; et al.	The first antitrust review of the patent settlement agreement
2013	In the case of *Actavis*	The US Supreme Court has made its first ruling on reverse payment agreements
2019	In the case of Reckitt Benckiser Group plc	The FTC brought its first filing against product hopping
2024	In the matter of Caremark Rx; Zinc Health Services; et al.	The FTC sues three of the largest pharmacy benefit managers
2001–2024	In the matter of Glaxo Wellcome plc/SmithKline Beecham plc	63 cases of regulating anti-competitive mergers and acquisitions
	In the matter of Pfizer Inc./Warner-Lambert Company	
	……	

**FIGURE 2:**
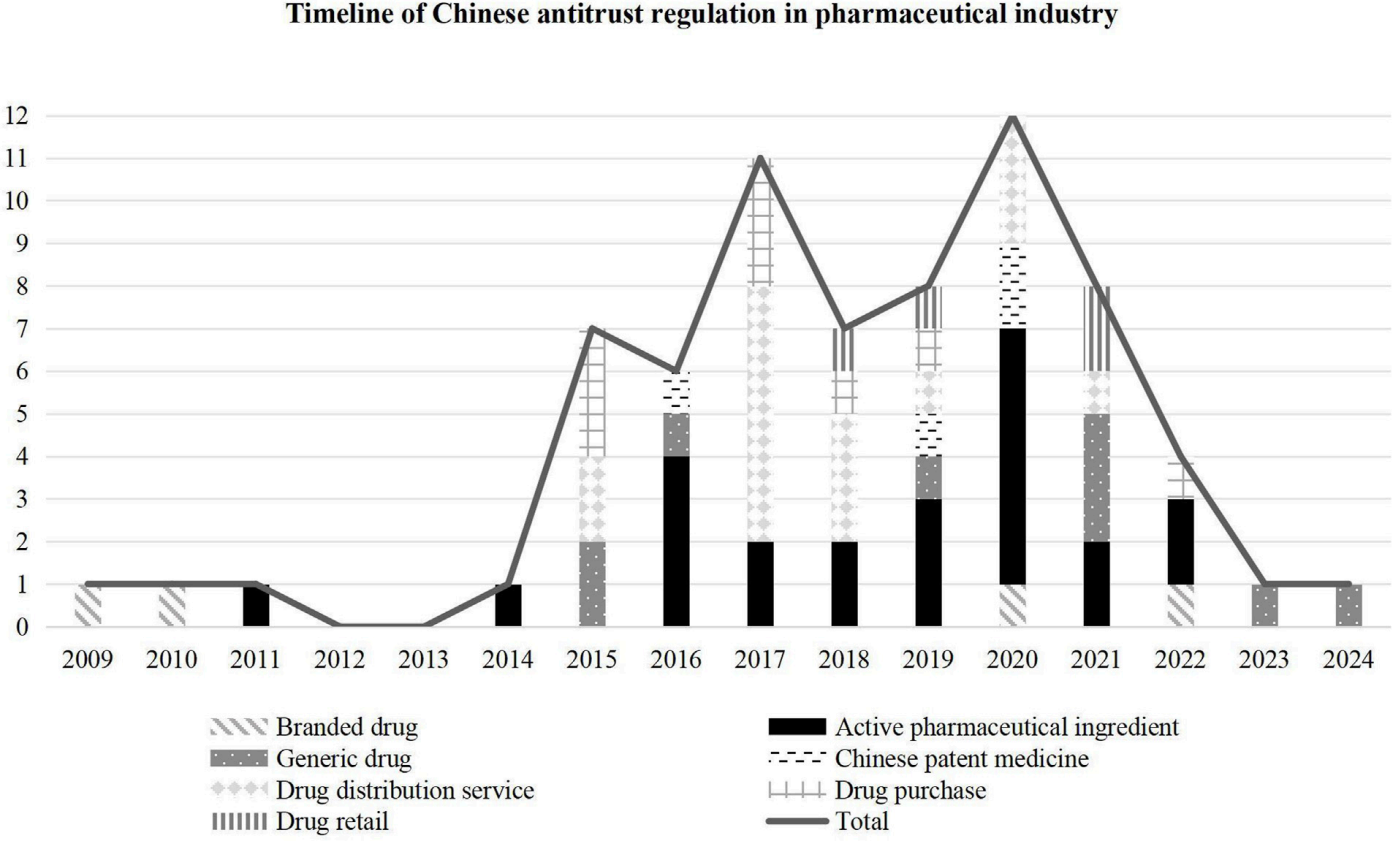
Timeline of Chinese antitrust regulation in the pharmaceutical industry (Based on filing time).

The timeline of Chinese pharmaceutical antitrust regulation reflected in Figure 2 can be divided into three stages:

Phase I: Pharmaceutical concerns in exploratory law enforcement. China formally issued the Antitrust Law in 2008. The three major agencies, the National Development and Reform Commission, the Ministry of Commerce, and the State Administration for Industry and Commerce, began exploratory law enforcement. The published cases involved an antitrust review of the pharmaceutical industry. In 2009 and 2010, the Ministry of Commerce reviewed two cases of operator concentration related to branded drugs. In 2011, the National Development and Reform Commission announced the first antitrust case of bulk pharmaceuticals, also called active pharmaceutical ingredients (API, active pharmaceutical ingredient). While implementing a centralized drug procurement policy in public hospitals (The state council the people’s republic of China, 2015a), some provincial administrative agencies abused their power to build up local protection, design transactions, and formulate regulations to exclude or restrict competition. As a result, the regulation of administrative monopolization in the centralized procurement and distribution management of medicines has become the focus of Chinese antitrust review since 2015.

Phase II: Focus on APIs in regular law enforcement. After introducing the fair competition review mechanism in industrial policy in 2016 (The state council the people’s republic of China, 2015b), the regulation of administrative monopolization has gradually become a regular area of enforcement. In 2017, it was found that this kind of case accounted for 70 percent. In 2019, the State Administration for Market Regulation (SAMR, State Administration for Market Regulation), which was established through the merger of three major antitrust enforcement agencies, announced a nationwide law enforcement campaign against targeting drug monopolies (The state council the people’s republic of China, 2019). The campaign focused on the monopolization of the API industry, which is strongly criticized by the public (The State Administration for Market Regulation, 2020). The API monopolization cases account for 46 percent of the total number of cases in the 4 years from 2019 to 2022.

Phase III: Specialized law enforcement in the field of people’s livelihoods. In February 2023, the SAMR announced that it would strengthen antitrust regulation in people’s livelihoods, including the pharmaceutical industry. In January 2025, this administration issued the Antitrust Guidelines in the Pharmaceutical Field, which means that the regulation will gradually extend to the whole chain of this industry.

Table 2 reflects changes in the regulatory focus and significant cases of Chinese pharmaceutical antitrust.

**TABLE 2:** Changes in the regulatory focus and significant cases of Chinese pharmaceutical antitrust.

Regulatory focus
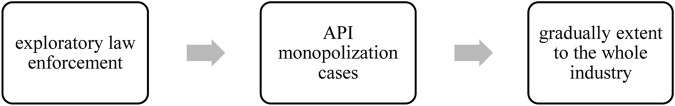

Significant cases		
Year	Cases	Description
2009	In the matter of Pfizer Inc./Wyeth	The first antitrust regulation in the pharmaceutical industry by the Ministry of Commerce
2011	In the matter of Shandong Shuntong/Shandong Huaxin	The first API antitrust case
2015	In the matter of Chongqing Qingyang; Chongqing Datong; et al.	The first price-related antitrust case
	In the matter of Anhui Bengbu	The first administrative monopolization case
2019	In the matter of Yangtze River Pharmaceutical Group	The first medicine antitrust case
2021	In the case of “Saxagliptin tablet”	The first antitrust review of the patent settlement agreement
2023	In the matter of Hoechst Marion Roussel, Inc./Carderm Capital L.P.; et al.	The first antitrust review of the patent settlement agreement
2024	In the matter of Shanghai Xinyi; Henan Runhong; et al.	The first antitrust case to both fine companies and individuals

### 3.2 Regulatory fields

**FIGURE 3:**
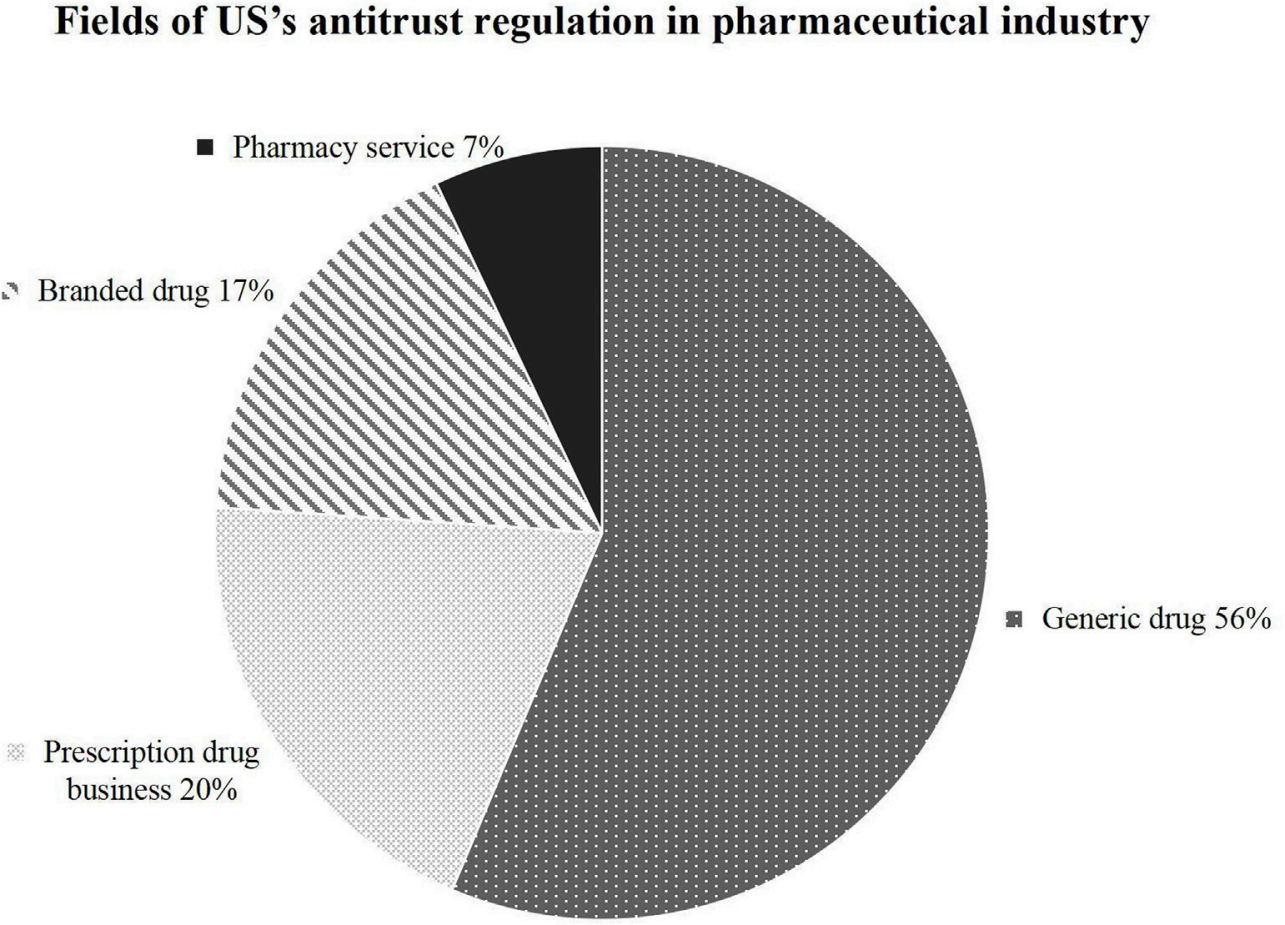
Fields of US’s antitrust regulation in pharmaceutical industry.

Analyzing the proportion of areas involved in United States’s pharmaceutical antitrust cases, as shown in Figure 3, the four major regions are characterized by strong innovation and return, and the proportion of branded and generic drugs belonging to pharmaceutical Research and Development (R&D, Research and Development) and manufacturing links has reached 74%. For this reason, after the Hatch-Waxman Act paved the way for generics to enter the market (Gary, 2019), drug prices fell by at least 20 percent within a year of the first generic drug entry (Berndt and Aitken, 2010), while five generics competing could drive nearly 85 percent of the price decline (FDA, 2016). Statistics show consumers saved at least $8 billion in 1994 by purchasing generic drugs (Federal Trade Commission, 2006). This figure soared to 217 billion in 2012, with total savings of $1.68 trillion between 2005 and 2014 (Feldman and Frondorf, 2016), which brought considerable consumer benefits. By 2016, the market share of generic drugs had increased from 19% in 1984 to 89%, and 90% of prescription drugs sold in the U.S. were generics (Gary, 2019). Therefore, the U.S.’s antitrust law enforcement focuses on the finished drug industry to promote competition between pharmaceutical companies to ensure further drug supply and reduce drug prices. Moreover, it indirectly encourages drug innovation intending to maintain the advantages of the U.S. pharmaceutical supply chain.

**FIGURE 4:**
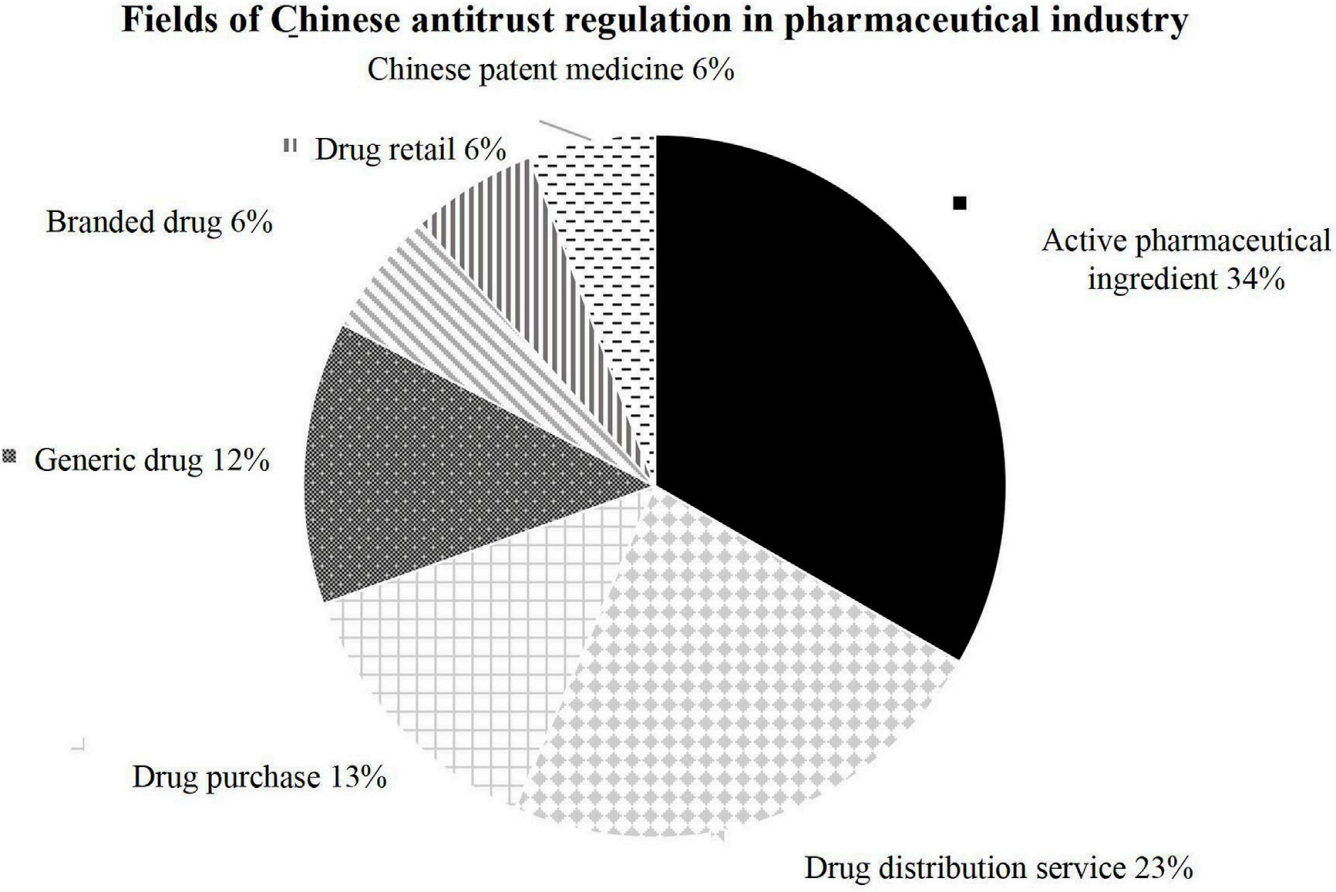
Fields of Chinese antitrust regulation in pharmaceutical industry.

The United States prioritizes competition in the finished drug market, while the Chinese regulatory focus turns toward the upstream API sector. This reflects the country’s unique industrial structure and policy objectives (as shown in Figure 4). The Chinese modern drug regulatory system started during rapid development of pharmaceutical technology and drug regulation worldwide. In 1950, China established the guideline of “focusing on the development of APIs and supplementing with preparations” (Zhao et al., 2024). Until the reform and opening-up, potential external and internal economies of scale and scope were not achieved. Most manufacturers relied on repetitive production of low-value-added bulk pharmaceuticals and imitation drugs (Neriman et al., 2019). By the 1990 s, due to environmental pressures, rising production costs, and other factors, the production capacity of APIs in Europe and the United States gradually declined, and the industry gradually shifted to developing countries with loose regulations and low costs. In the 21st century, China and India supply more than 80 percent of the bulk pharmaceuticals used to produce prescription drugs in the U.S. (Gary, 2019). China had about 7,000 base ingredient manufacturers in 2019 (Neriman et al., 2019), which stands out as the world’s largest API supplier. It can be said that the Chinese modern pharmaceutical industry developed passively and rapidly under the impact of western industry and economic globalization. Since implementing the Antitrust Law, China has focused its antitrust regulation on the API industry first to enhance its supply chain, which has been developing for decades. Since the 12th Five-Year Plan (2011–2015), the Chinese government has shifted its strategy toward incentivizing innovation (Sandra, 2024), and firms manufacturing branded drugs and Chinese patent medicine are also under antitrust regulation.

In the United States, antitrust enforcement mainly targets the finished drug market, where competition between branded drugs and generics promotes price reduction. Key mechanisms include the regulation of patent settlements and merger reviews under the Hart-Scott-Rodino Act. In contrast, Chinese regulatory focus on the upstream sector to address monopolistic pricing and supply chain bottlenecks. This prioritization reflects China’s role as the world’s largest API supplier and the government’s consideration of public welfare.

### 3.3 Types and behaviors of pharmaceutical antitrust violations

**FIGURE 5:**
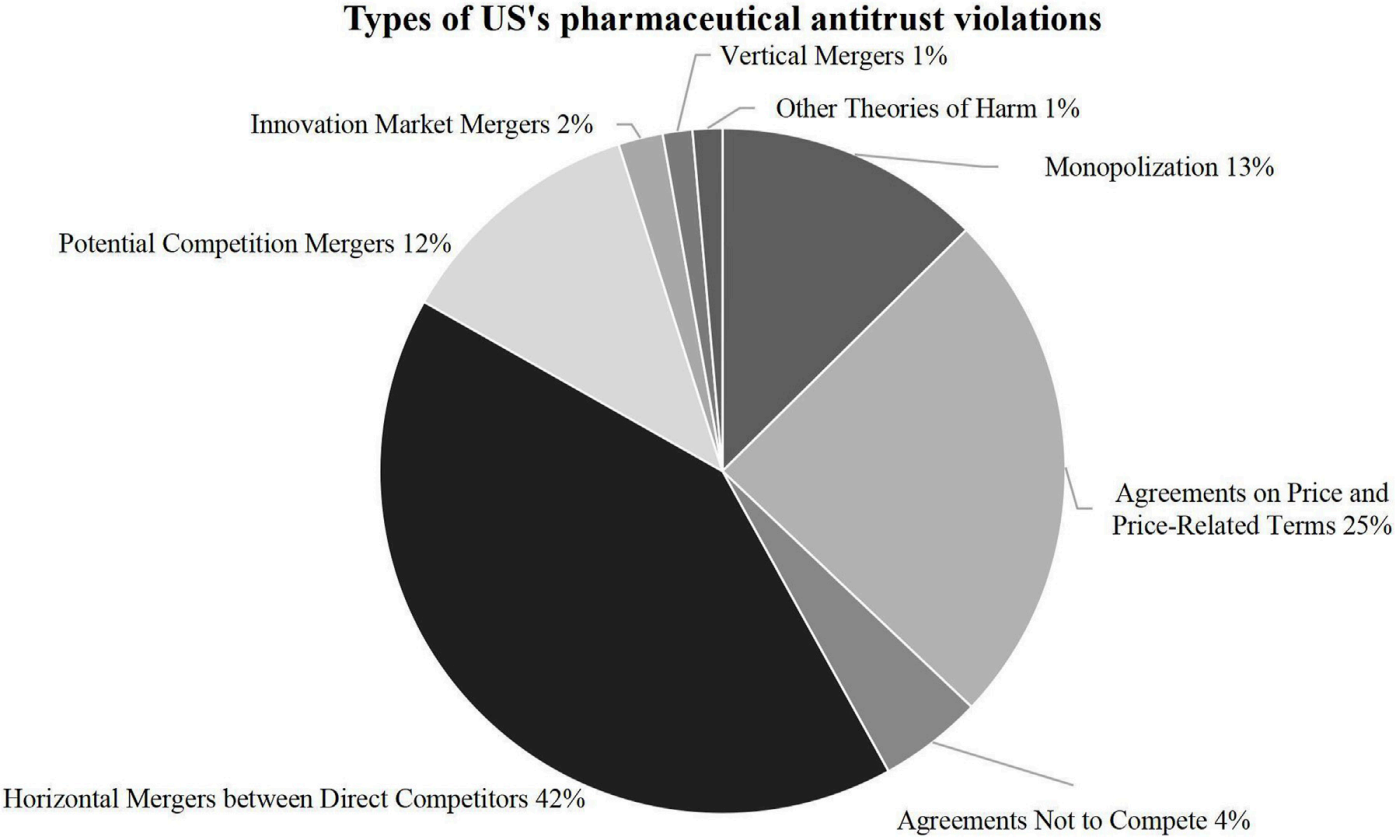
Types of US’s pharmaceutical antitrust violations.

**FIGURE 6:**
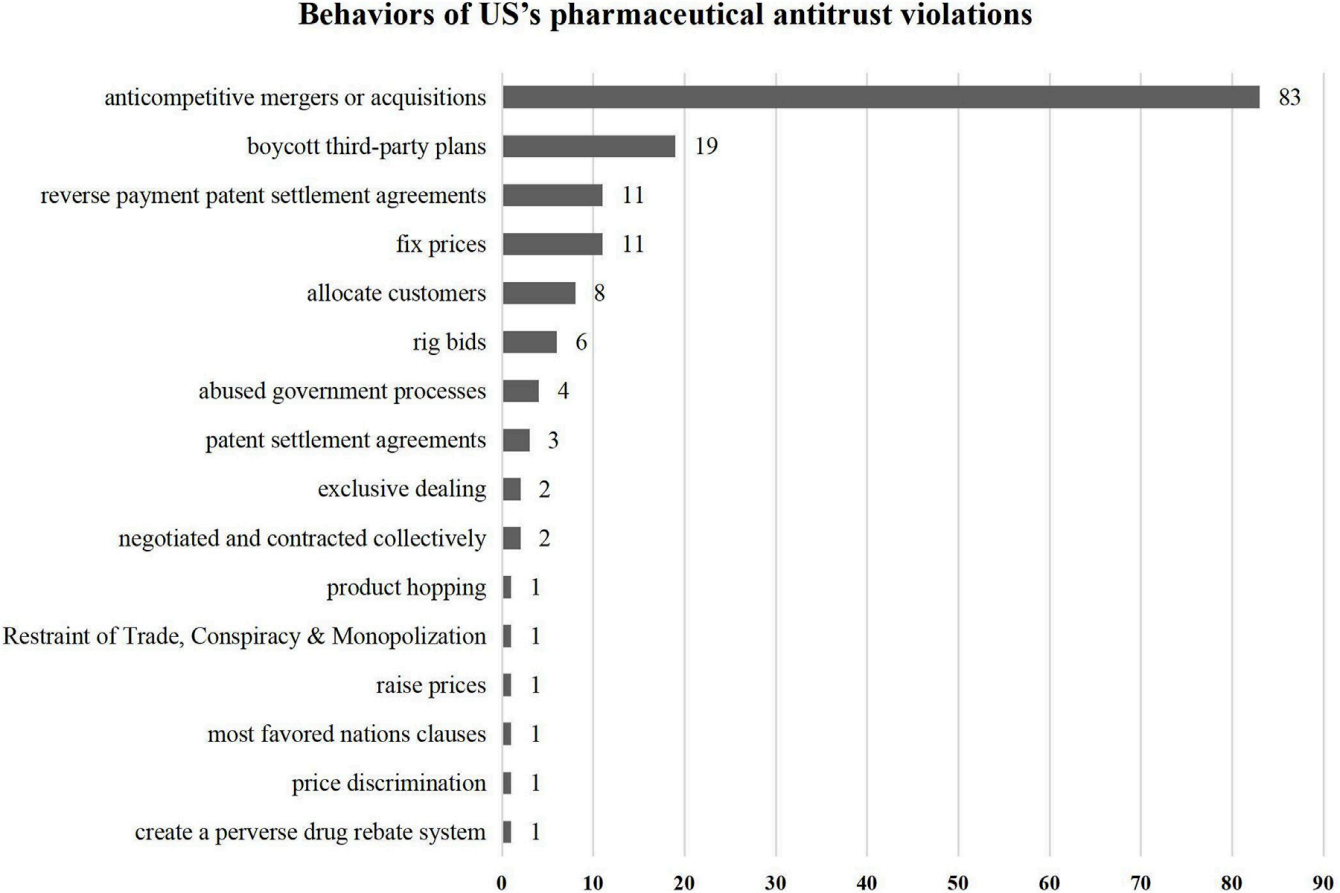
Behaviors of US’s pharmaceutical antitrust violations.

This article refers to the overview of FTC actions in pharmaceutical products and distribution issued in October 2024 to classify the types of pharmaceutical antitrust cases as follows (Figure 5). It provides statistics on specific behaviors (Figure 6). The study found that pharmaceutical antitrust in the United States regulates a wide range of anti-competitive mergers and acquisitions, and the number far exceeds that of another type of cases. This type is typically characterized by “killer acquisitions,” that is, existing companies seize the opportunity of future competition by acquiring innovation targets and terminating their research program (Colleen et al., 2021) or to restrict competition to seek expected increases in profits and investments (Congressional Budget Office, 2021). For the branded drug market, less competition may affect innovation, new drug approvals and many other factors across the industry (Gary, 2019). Moreover, this kind of killer acquisition can also be used to describe the mergers and acquisitions behavior of the generic market. Major generic pharmaceutical companies obtain market dominance through anti-competitive market concentration, which impedes low-cost generics’ survival. Another highlight of the United States’s pharmaceutical antitrust is that, in addition to controlling general price-fixing agreements, it provides practical experience in regulating new forms of monopoly acts in the type of “monopolization”. For example, there have been abundant cases of “reverse payment agreements” and precedents for enforcement of false patent infringement suits, product hopping and patent evergreen, and Sham Orange Book Listing.

**FIGURE 7:**
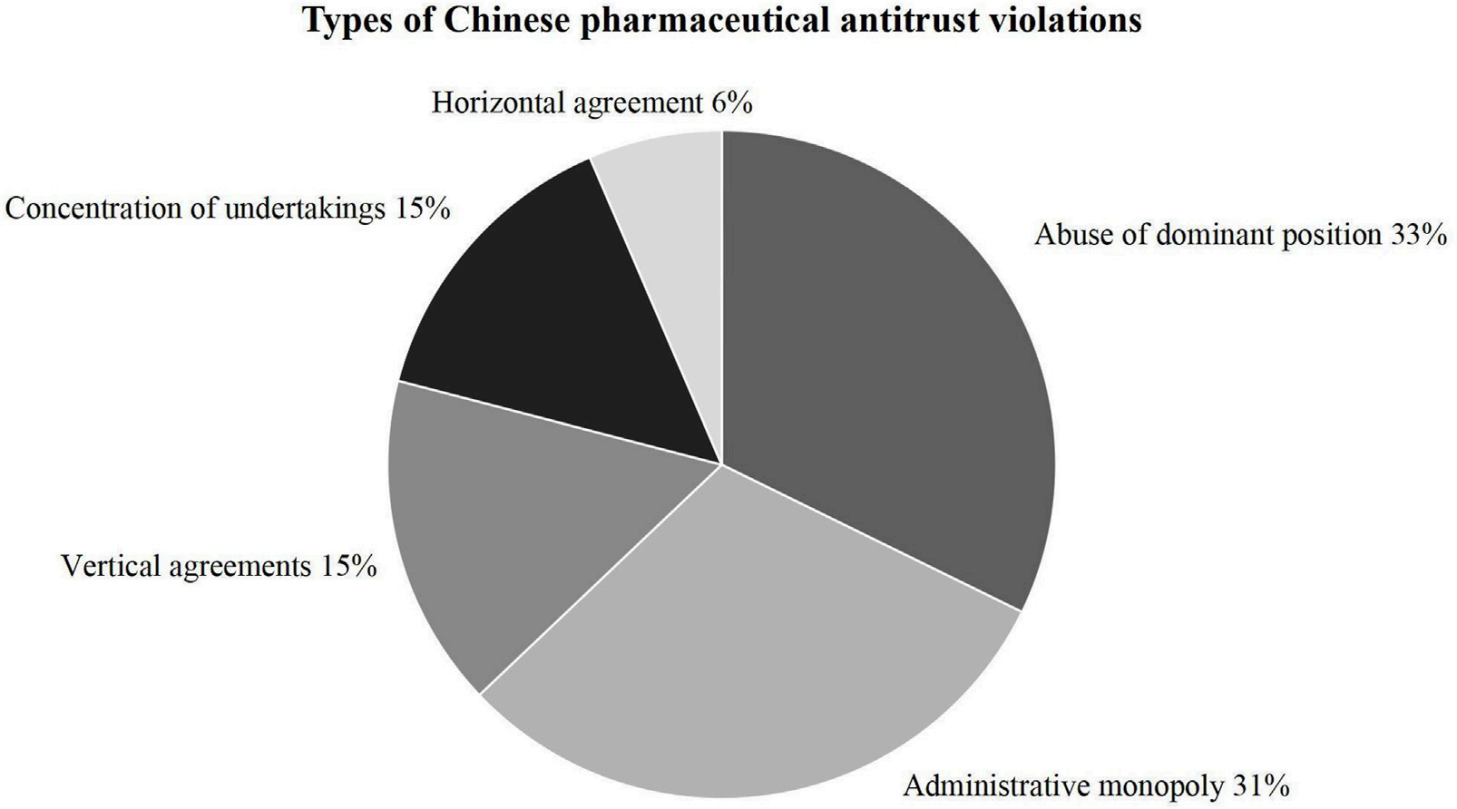
Types of Chinese pharmaceutical antitrust violations.

**FIGURE 8:**
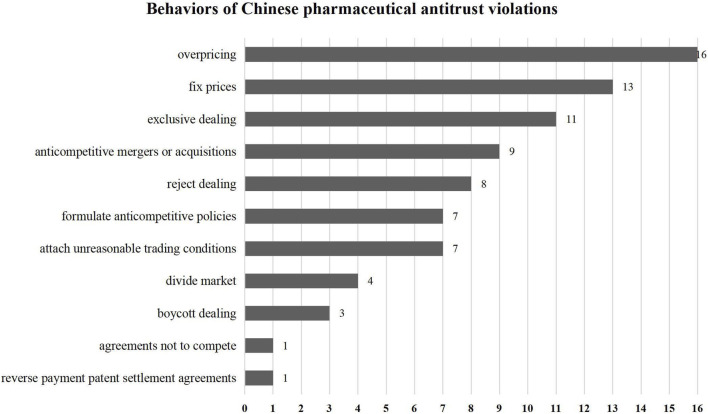
Behaviors of Chinese pharmaceutical antitrust violations.

The classification of the Annual Report on Antitrust Enforcement in China is consistent with the three types of economic monopolization and one type of administrative monopolization regulated by the Antitrust Law. As shown in Figure 7 and Figure 8, cases of operator concentration account for only 15 per cent, and 21 percent are related to monopoly agreements. By contrast, abuse of dominant positions accounted for the largest share, up to 33 percent. Notably, the enforcement activities to regulate administrative monopolization amounted to 31 percent in the pharmaceutical industry. It is found that pharmaceutical antitrust in China still mainly focuses on obvious and easier to identify behaviors, such as overpricing, rejecting dealing and other behaviors that directly affect the drug price. Affected by the nature of the API industry, there is usually only one or several suppliers in the market thus unreasonable price increases are more straightforward to observe and recognize. Although monopoly behaviors have also occurred in the branded drugs and generics markets recently, the number of innovative companies in China is relatively small. And there exists an ample space for development, determining monopoly behavior is nothing more than the concentration of foreign operators or a single pharmaceutical manufacturer implementing monopoly behavior. Chinese antitrust law in the pharmaceutical industry mainly maintains competition and has not yet been able to stimulate innovations fully. However, it is worth affirming that monopolization in China includes administrative monopolies. Unlike the developed commercial health insurance in the U.S., commercial health insurance in China is relatively limited in total national healthcare expenditure (CBIMC, 2024). Therefore, the Chinese government plays a significant role in reducing healthcare costs. Government participation means that the antitrust law also needs to regulate the behavior of administrative agencies that exclude or restrict market competition.

## 4 Discussion on the antitrust roads in the United States and China

### 4.1 Principles of regulation

**FIGURE 9:**
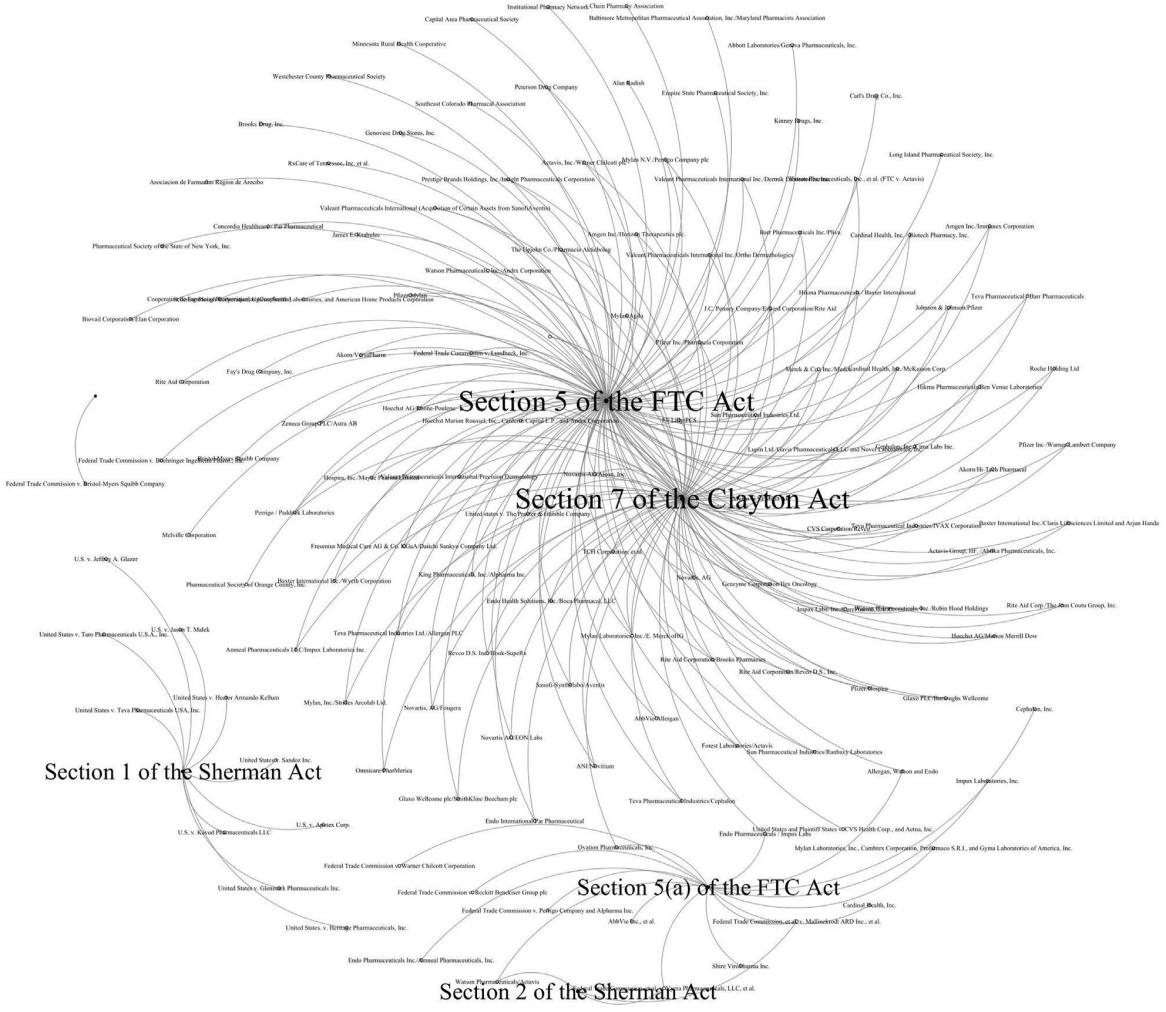
Major statutory basis of US’s pharmaceutical antitrust violations.

In the research process, we noticed that the principles of pharmaceutical antitrust regulation in the United States and China present the difference between abstract and concrete regulation. To analyze these two roads further, we extract the specific bases for determining violations in the U.S. and Chinese antitrust law enforcement documents and form the following visualization figures with “case name-legal basis” as the node. As shown in Figure 9, the primary legal bases for antitrust violations in the U.S. pharmaceutical industry are Sections 1, 2 of the Sherman Act, Section 7 of the Clayton Act, and Section 5 of the FTC Act. These articles have not specified monopoly behavior since the first introduction in 1890 and 1914. As a frequently used clause of American antitrust law, Section 1 of the Sherman Act provides that “Every contract, combination in the form of trust or otherwise, or conspiracy, in restraint of trade or commerce among the several States, or with foreign nations, is declared to be illegal”; Section 2 provides that “Every person who shall monopolize, or attempt to monopolize, or combine or conspire with any other person or persons, to monopolize any part of the trade or commerce among the Several States… shall be guilty of a felony … “. However, the legislation does not explicitly restrict trade and specific manifestations of monopolistic behavior. Section 7 of the Clayton Act merely prohibits mergers and acquisitions where the effect “may be substantially to lessen competition or to tend to create a monopoly”, without specifying the specific considerations in the review. Section 5 of the FTC Act bans “unfair methods of competition” and “unfair or deceptive acts or practices,” without even further defining what “unfair” means. Abstract and highly generalized provisions of the U.S.’s antitrust laws make enforcement more adaptable and flexible. In specific cases, law enforcement agencies and federal courts apply those provisions combined with review rules such as the “*per se* illegal rule” and the “rule of reason.”

**FIGURE 10:**
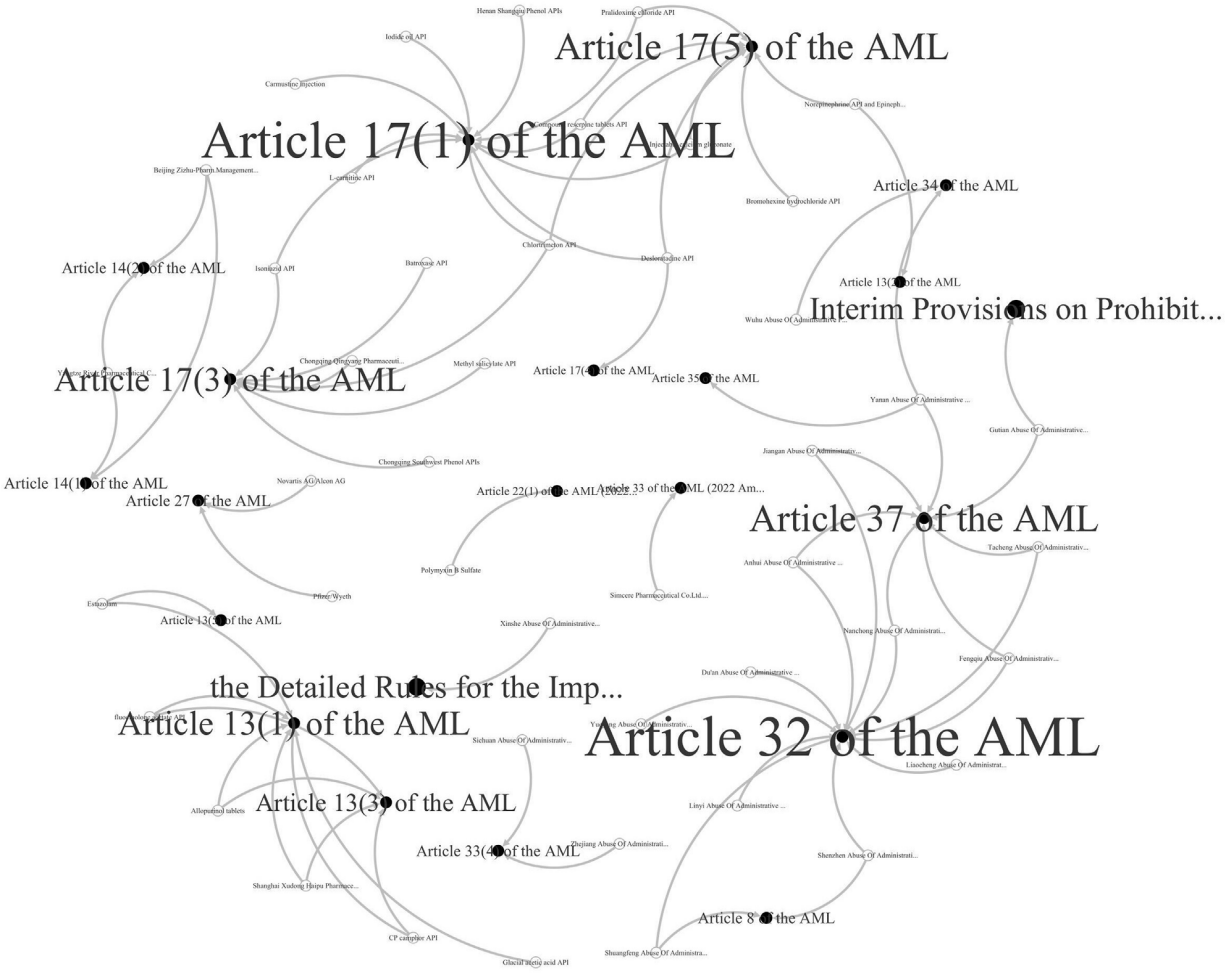
Major statutory basis of Chinese pharmaceutical antitrust violations.

The same methodology was used to analyze the 60 Chinese cases collected, resulting in the Figure 10 visualization. Different from the six significantly aggregated nodes in Figure 9 above, there are more scattered nodes in Figure 10 with fewer relevant cases. The Antitrust Law in China, along with related guidelines, enumerates a legal basis specific to antitrust law enforcement. For example, compared to the Sherman Act, article 13 of the Antitrust Law lists at least six forms of agreements in restraint of competition, and article 17 frequently applied provides at least six forms of monopoly behavior. Furthermore, articles 32 and 37 are the legal basis for administrative monopolization. As a statutory law country, the concrete, strives for antitrust regulation with laws to follow and can regulate monopoly behavior accurately and efficiently.

### 4.2 Means of regulation

As a case law country, the uncertainty of antitrust legislation in the United States enables the federal courts to interpret antitrust law by the standard law model and to develop a set of rules to make businesses and markets operate in a socially efficient manner (Herbert, 2005). Without exception, pharmaceutical antitrust enforcement in the United States is also judicially driven. Specifically, in addition to reviewing lawsuits filed by the DOJ and remedies or injunctions sought by the FTC, the courts also take a leading role in establishing pharmaceutical antitrust deterrence and exploring review rules. Since the Supreme Court determination in the case of FTC v. Actavis, potential pay-for-delay patent dispute settlements entered into by pharmaceutical companies decreased significantly in fiscal year 2014; the total number of such deals filed with the FTC has dropped to 21 in Fiscal Year 2014 from 29 in Fiscal Year 2013, and 40 in Fiscal Year 2012 (Federal Trade Commission, 2016). Following the *Actavis* ruling and subsequent case law, the use of the type of reverse payment agreement, which most likely to harm consumers, continued to decline (Federal Trade Commission, 2020). Meanwhile, in *Actavis*, the Supreme Court returned to the rule of reason in its review of reverse payment agreements, which had previously been analyzed by the *per se* illegal rule applied by the Sixth Circuit in Andrx in 2001; the scope of the patent test by the 11th Circuit in Schering-Plough in 2003, and the quick look rule applied by the Third Circuit in K-Dur in 2012 (Chen, 2018). Generally speaking, judicially-driven antitrust regulation will strictly follow legal procedures with the imposition of criminal or civil liability. However, over half of the collected cases ended with a “consent order”. In a consent order, the defendant does not have to admit committing monopolistic conduct but to give undertakings to the FTC, including ceasing the illegal conduct, abandoning certain rights that may restrict competition in the market, compensating the plaintiff and so on. On the one hand, these consent orders shall be recognized and executed by the court; on the other hand, both parties of an order are the defendant and the law enforcement agency, which also exposes the feature of administrative agreements. With the help of contractual cooperation mechanisms and the modesty of flexible enforcement, antitrust law-enforcement settlement can adapt to innovation, and thus extend to more market fields, and cover new market behaviors. It also makes up for the defects of rigid tools (Zhan, 2023).

Chinese pharmaceutical antitrust enforcement is administratively led, whether through the division of responsibilities among the National Development and Reform Commission, the Ministry of Commerce and the State Administration for Industry and Commerce before 2018, or the SAMR taken up the unified antitrust enforcement since 2018, the antitrust law enforcement mainly in the form of making decisions on penalties by administrations. Among the 61 Chinese pharmaceutical antitrust cases, 54 cases were regulated by the above law enforcement agencies. In addition, current Chinese pharmaceutical antitrust enforcement tools are still based on rigid enforcement. There are only two administrative cases in which the investigation was discontinued due to the operator’s commitment to rectify the situation, in contrast to the rest which resulted in high fines. Although the Antitrust Law contains flexible enforcement measures, operator commitment similar to that of a consent order is rarely applied.

### 4.3 Effects of regulation

Firstly, regarding the direct effects of regulation, different regulatory approaches in the United States and China have helped lower drug prices. As shown in Table 3, the statistics on the competition situation in the pharmaceutical market of the collected cases; the pharmaceutical antitrust mainly focuses on the drugs in shortage, and some even have no more than three manufacturers. The fewer suppliers in the market, the more likely it is to increase the price (Marc and Karena, 2017). For this reason, antitrust enforcement restores competitive market prices for drugs by cracking down on monopoly prices set by suppliers, and further reducing the cost of patients and national health insurance expenditures. Public information shows that China promoted the price reduction of Polymyxin B by nearly 90 percent, from 2,303 to 2,918 yuan to 270 yuan (China Competition Law and Policy, 2023).

**TABLE 3:** Statistics of market competition in both US’s and Chinese pharmaceutical antitrust cases.

Statistics of market competition in both US’s and Chinese pharmaceutical antitrust cases		
Countries Drugs	The United States	China
Number of drugs which only one manufacturer	Branded drug: 9 Generic drug: 33	API: 4 Branded drug: 1 Generic drug: 1 Chinese patent medicine: 4
Number of drugs which no more than three manufacturers	Branded drug: 22 Generic drug: 66	API: 9 Branded drug: 1
Number of drugs which no more than ten manufacturers	Branded drug: 4 Generic drug: 42	API: 8 Generic drug: 3 Chinese patent medicine: 1
Other drugs in shortage	Branded drug: 13 Generic drug: 185	API: 1 Branded drug: 2 Generic drug: 1

Secondly, the U.S.’s pharmaceutical antitrust regulates the new market that the Hatch-Waxman Act creates. The market competition mainly happens between brand drugs and their generics, resulting from monopoly profits brought by patent protection periods and technical barriers. Pharmaceutical antitrust in the United States creates more competition by requiring monopolists to abandon their patent rights or to transfer technology for drug development. Expressly, in patent settlement disputes, agencies prohibit generic companies from delaying the introduction of their drugs whereas they require the branded companies to license the right to manufacture synthetic alternatives. In mergers and acquisitions cases, the acquirer must divest some drug production lines or transfer the right to manufacture some drugs. Table 4 presents a non-exhaustive count of new competitors entering relevant markets in the U.S.’s pharmaceutical antitrust regulation.

**TABLE 4:** New competitors entering relevant markets in US’s pharmaceutical antitrust regulation.

New competitors entering relevant markets in US’s pharmaceutical antitrust regulation		
Types Drugs	Branded Drug	Generic Drug
Number of new competitors entering relevant markets in drugs which only one manufacturer	7	20
Number of new competitors entering relevant markets in drugs which no more than three manufacturers	23	34
Number of new competitors entering relevant markets in drugs which no more than ten manufacturers	4	14
Number of new competitors entering relevant markets in other drugs in shortage	5	22

In the United States, antitrust remedies for injured parties are more direct. The most typical example is monetary remedies. Section 13b of the FTC Act enables the FTC to seek a permanent injunction, on which the agency frequently requires the operator to provide financial compensation for injured consumers in a settlement order. As the most potent tool, has provided billions of dollars in relief to consumers over the past 40 years (Federal Trade Commission, 2021). In a pharmaceutical antitrust dispute, the FTC reached a $100 million monetary settlement as early as 1998 in the Mylan Laboratories, Inc. The case of Cardinal Health, Inc. in 2015 was the second-largest monetary settlement. Moreover, 97.8 percent of the monetary remedy returned to more than 50,000 consumers in the case of Reckitt Benckiser Group plc (Tableau, 2020).

Chinese pharmaceutical antitrust has regulated the existing market since the development of the modern pharmaceutical industry, with the supply of the API as the main advantage. The production of APIs is subject to strict production authorization, resulting in a small number of manufacturers (Li and Li, 2019). Besides, some pharmaceuticals, such as Methyl salicylate API, is produced by even rare companies due to the pollution effect, energy consumption and low profits (Hubei Industry and Commerce Administration, 2017). Due to their unique nature, some APIs and their downstream preparations are still in short supply. For example, the monopolization of allopurinol tablets in 2015 and isoniazid tablets in 2017, are currently in shortage. In contrast to the direct remedy for consumers in the United States, damaged consumers in Chinese pharmaceutical antitrust can only seek relief by filing private lawsuits or through public interest litigation by Article 60 of the Antitrust Law.

However, it is worth noting that the penalties in China are rather high. In the Yangtze River Pharmaceutical Group monopolization case, the SAMR issued a fine of up to Chinese Yuan (CNY, Chinese Yuan) 760 million against a single company (State Administration for Market Regulation, 2021). In this study, we analyze the subsequent business performance of the four pharmaceutical companies subjected to the most stringent antitrust administrative penalties in China, as shown in Table 5. The data reveals a significant decline in annual revenue and net profit for some companies immediately following penalties, followed by a gradual recovery to a growth trend by the third year. A representative case is Yangtze River Pharmaceutical Group, whose revenue declined to ¥78.553 billion in 2021 (a 21.9% year-on-year decrease) but increased to ¥78.285 billion in 2023, marking a 194.02% year-on-year surge. In addition, the companies involved in the case generally adjusted their business strategies after the antitrust regulation. They gradually shifted their goals to improve innovations based on the branded drugs to break through the patent barriers. In the case of Simcere, for example, its innovative drug revenue share increased significantly from 62.4% in 2021 to 74% in 2024 (Simcere, 2024).

**TABLE 5:** The operation of some companies after Chinese antitrust administrative punishment in the pharmaceutical industry.

Penalty effect Companies	Amount of penalty (CNY)	Year of penalty	Annual revenue after penalty (CNY)	Annual profit after penalty (CNY)	Number of new drugs
Yangtze River Pharmaceutical Group	764 million	2021	• Year 2021: 78.553 billion (21.9% decline) • Year 2022: 26.626 billion (66.1% decline) • Year 2023: 78.285 billion (194.02% increase)	Undeclared	• Year 2021: 3 Innovative drugs, 52 generics • Year 2022: 4 Innovative drugs, 39 generics • Year 2023: 2 Innovative drugs, 28 generics
Simcere	100.7 million	2021	• Year 2021: About 5 billion (10.9% increase) • Year 2022: 6.319 billion (26.4% increase) • Year 2023: 6.608 billion (4.6% increase) • Year 2024: 6.635 million (0.4% increase)	• Year 2021: 1.499 billion (125.6% increase) • Year 2022: 933 million (37.76% decline) • Year 2023: 700 million (3.6% increase) • Year 2024: 733 million (4.71% increase)	• Year 2021: 11 innovative drugs • Year 2022: 4 innovative drugs • Year 2023: 7 innovative drugs
Grand Pharmaceutical Group Limited	285 million	2023	• Year 2023: About 9.8 billion (15.8% increase) • Half of Year 2024: About 5.6 billion (1% increase)	• Year 2023: 1.903 billion (0.2% increase) • Half of Year 2024: 1.455 billion (51.4% increase)	• Year 2023: 30 products on the market, 5 innovative drugs
NORTHEAST PHARM	133 million	2023	• Year 2023: 8.243 billion (6.42% decline) • Half of Year 2024: 4.169 billion (7.5% decline)	• Year 2023: 358 million (2.34% increase) • Half of Year 2024: 278 million (46.05% increase)	• Year 2023: 6 innovative drugs

Through high fines and strict punishment measures, the Chinese pharmaceutical antitrust enforcement mechanism not only effectively curbed the monopolistic behavior of the enterprises involved, but also performed a significant deterrent effect on other entities in the industry. From the point of view of the institutional effect, this kind of administrative penalty has the following functions: one is to recover the competition order by correcting market failure, such as forcing companies involved to abuse price-fixing agreement and to reduce prices in a short time; the other is to guide the companies to restructure development strategy and to promote resources to the field of R&D. While optimizing the efficiency of market resource allocation, this dual mechanism of “discipline - guidance” also ensures the stability of API supply chain, and finally realizes patient welfare by reducing the production cost of downstream preparations. Furthermore, it also pushed the National Health Insurance Bureau to clamp down on drug monopoly control. Since 2023, 23 companies involving 30 varieties have been interviewed, and the average price reduction of the interviewed drug has exceeded 40 percent (News, 2024).

## 5 Discussion and recommendation

The above analysis shows that the U.S. pharmaceutical industry antitrust regulation has more practical experience, an important reference for China. Drawing lessons from the U.S. regulation can effectively improve Chinese pharmaceutical antitrust statute law and law enforcement. It can be seen that the U.S. and China are different in terms of cultural background, antitrust legal system, and market maturity. “Healthy competition equals healthy consumers,” the American free-market system is built on the premise that open competition and consumer choice maximize consumer welfare. The same is true for the pharmaceutical industry with a complex industrial chain. Thus, the FTC and the DOJ enforcement actions protect the free market system from anti-competitive behavior and promote market innovation (Federal Trade Commission, 2004). In the Chinese context, “consumer welfare” is usually included in the value of “people’s livelihood” due to the transformation from a pre-reform command economy. The main problems faced by the Chinese current market economy are administrative monopoly, state-owned, and even foreign monopolies (Li, 2011). Therefore, people’s livelihood includes the protection of individual welfare and the overall social welfare under government control. Consequently, we can only effectively restrain the government and the market. However, in conclusion, the common goal of both countries is to ensure that consumers have access to low-priced, adequate supply of drugs and services. In the background of value convergence, this article puts forward the following suggestions for the further development of Chinese pharmaceutical antitrust regulation.

### 5.1 Emphasizing principle and precaution

Under the comparative law perspective, the “principle and precaution” of antitrust regulation is embodied in the different choices of legal system for maintaining market competition order. As a statutory law country, the Chinese antitrust framework is characterized by a precise definition of behavior elements. Its normative text enumerates monopoly agreements and abuse of dominant market position by types through Articles 17 and 18 of Antitrust Law. Although this legislative technology strengthens the certainty of law application, it is difficult for law enforcement agencies to regulate potential anti-competitive behaviors. In pharmaceuticals, antitrust enforcement agencies should be more active in identifying and controlling anti-competitive schemes before patients are harmed (American Economic Liberties Project, 2023). In contrast, as a typical case law country, the United States has more flexibility in interpreting and applying abstract antitrust provisions in its practice. Therefore, acquiring insights from the U.S.’s methodology, although abstract rules cannot be directly applied in China, they can be used as overall law enforcement principles to establish an antitrust preventative mechanism.

Article 37 of the Antitrust Law, amended in 2022, explicitly requires the State Council to improve the hierarchical classification-based examination system of operators involved in important sectors such as people’s livelihood. Moreover, the Antitrust Guidelines in the Pharmaceutical Field have also clarified five regulatory enforcement principles, with the innovative introduction of a compliance guideline system. Therefore, China has established a preliminary normative framework for establishing a pre-regulatory system for pharmaceutical antitrust. In the future implementation stage, Chinese antitrust law enforcement agencies can gradually bring the whole chain of the pharmaceutical industry into the scope of regular supervision under the guidance of the legislative spirit, and construct specific prevention mechanisms from the perspectives of system design and innovation of law enforcement tools according to characteristics of the pharmaceutical industry.

Firstly, classifying and grading Internet platforms can be referred to as categorizing pharmaceutical companies into high, medium, and low-risk levels and implementing differentiated regulatory strategies (Ma, 2023). For example, dynamic monitoring can be implemented for manufacturers of short supply, key materials and large-scale pharmaceutical enterprises to detect and regulate pharmaceutical monopoly behaviors. Comparatively, compliance training can be given priority to small and medium-sized generic pharmaceutical companies to reduce regulatory costs. At the same time, according to the characteristics of R&D, production, circulation and other links, classified policies can be adopted: the research end focuses on preventing “patent jungle” and reverse payment agreements; the production side strengthens the obligation to data disclose of API production capacity, and the circulation end cracks down on regional marketing control alliance. Secondly, we can make good use of the antitrust enforcement action in the livelihood field to pay timely attention to the problems of excessive drug prices, drug shortages and other monopoly risks that reflected by the public. Meanwhile, it is worth carrying out antitrust compliance training to enhance the awareness and ability of pharmaceutical operators (State Administration for Market Regulation, 2024).

### 5.2 Coordinating innovation and regulation

Unlike the United States, which mainly adopts soft means such as consent orders, Chinese pharmaceutical antitrust law enforcement exhibits a rigid restraint landscape characterized by strict punishment procedures, strong deterrence and higher fines. Still, it fails to solve the problem of insufficient drug supply fundamentally. Chinese pharmaceutical market has not gone through the process from free competition to the gradual emergence of monopoly, and due to the inadequate protection of intellectual property rights in the early years, the domestic brand drug market protection level is relatively low, making the existing foreign generics and off-patent brands to dominate the domestic market (Neriman et al., 2019). Not until China amended the Antitrust Law in 2022 and the antitrust guidelines in the pharmaceutical sector did the goal of encouraging innovation written in antitrust law. It is, therefore, imperative to coordinate the encouragement of innovation and antitrust regulation.

In terms of encouraging innovation, since the drug review and approval reform in 2015, Chinese enterprises have shown increasing focus on innovative drugs. The innovative drug industry entered a period of rapid development in 2020 and achieved a breakthrough growth in the number of products under research in 2024 (Chen et al., 2024). However, compared with the mature pharmaceutical market in the United States, the Chinese pharmaceutical market still lacks sufficient endogenous inspiration, and the market concentration is low. Therefore, rather than mechanically adopting the U.S. approach of prioritizing the entry of numerous small and medium generics manufacturers, China should focus on supporting large pharmaceutical companies with strong market dominance to advance R&D. This entails not only speeding up the drug-testing cycle, reducing the authorization process and liberalizing pharmaceutical joint ventures but also requires that antitrust regulation to a certain extent, observe the principle of proportionality. For instance, in implementing precaution mechanisms, it is unnecessary to adopt stringent enforcement standards but rather to apply the operator commitment system flexibly according to the severity of the behavior, the market contribution, and the degree of compliance (Ma, 2023). This effort aims to foster continuous innovation in the pharmaceutical market through flexible law enforcement measures, to create more excellent social benefits for consumers and society to compensate for the negative impact of monopoly behavior. During ex-post regulation, a coordination mechanism of three aspects of “intergovernmental coordination–government and enterprise coordination - industry self-discipline” can be constructed. Firstly, through the inter-departmental joint conference involving the SAMR, the National Medical Products Administration, and the National Intellectual Property Administration, priority review for innovative drugs can be linked with antitrust compliance. Companies that meet specified R&D investment thresholds can be granted the application of the antitrust exemption system. Secondly, for dominant pharmaceutical companies engaged in monopolistic practices, reference can be made to the specific measures of the United States consent order, which allows them to promise to open up their patent pools or set up a fund for the R&D of generics in exchange for a reduction in the administrative penalties. Thirdly, it is also necessary to give full play to the role of industry associations by implementing compliance guidelines and enhancing industry self-regulation.

### 5.3 Enhance the importance and involvement of the judicial antitrust function

The function of judicial is to establish fundamental adjudicative rules. However, in practical implementation, China adheres to an administrative enforcement dominant antitrust regulatory model. Therefore, strengthening judicial antitrust function does not signify transitioning to a court-led system but rather to serve the functions of the antitrust enforcement agencies. Since 2021, the Supreme People’s Court of China has commenced issuing representative antitrust cases. By 2022, enhancing antitrust judicial governance has been formally incorporated as a key work objective, with the first antitrust review case in the pharmaceutical industry being initiated accordingly. Currently, of the 61 pharmaceutical antitrust cases in China, only six are reviewed by courts, and four of them only stay at the procedural level because of jurisdictional issues. What is more, except for one case of administrative inaction, the rest are all regarding contractual disputes. Chinese judicial antitrust function has not been fully exerted. Given the great importance of the pharmaceutical industry to consumers, it is necessary to enhance the importance and involvement of the judicial antitrust function. This study suggests that improvements can be made in private and public interest litigations under Article 60 of the Antitrust Law:

Article 60 of the Antitrust Law allows individuals who suffered damage from monopolistic behavior to sue for compensation. However, as ordinary people, consumers are often in a vulnerable position, with limited information and insufficient motivation to sue. Hence, referring to the incentive system of triple compensation in the United States, private litigation can be encouraged by allowing victims to claim triple compensation for monopoly behaviors. At the same time, in pharmaceutical antitrust civil litigation, the burden of proof on consumers as plaintiffs is appropriately reduced, and only the existence of monopolistic behavior and the fact of damage need to be proved, with the defendant bearing the burden of proof of the legitimacy of competition. Regarding public interest litigation, which is newly introduced into the Antitrust Law, the procuratorial organs should play a more active supervisory role in the antitrust judiciary. It is suggested to explore the application of punitive damages, providing procuratorial recommendations and other legal measures (Wu, 2024). By doing so, it forms a system of coordination with antitrust administrative enforcement and private litigation to achieve the legislative goals of the antitrust law.

## 6 Conclusion

The study’s findings reveal distinct characteristics in antitrust regulation for the pharmaceutical industry in the United States and China. First, regarding the regulatory status quo reflected in the cases, the United States demonstrates the flexibility to identify and deter monopolistic conduct in an intensely competitive market for finished pharmaceutical products. In contrast, China exhibits the characteristic of strictly penalizing prominent monopoly behavior in a pharmaceutical market with insufficient competition. Secondly, the two countries’ regulatory paths reveal the differences between legal systems and industry development. The United States, with its market-oriented commercial insurance system, requires antitrust regulation to balance pharmaceutical innovation and price competition. Its regulatory approach emphasizes timely adaptation to market changes while fostering market competition in the long term. In contrast, China is based on the public health insurance system, and antitrust regulation needs to prioritize access to drugs and the stability of supply. The Chinese model primarily employs rigid regulatory constraints to restore market order, aiming to promote the price to return to the reasonable range as soon as possible. Despite institutional differences, both countries have demonstrated that the ultimate goal of antitrust regulation is the protection of public health and that communication and cooperation between the two countries in the antitrust field give the possibility of mutual learning. Therefore, based on the results of the comparative study, we put forward the following suggestions for the future development of pharmaceutical antitrust regulation in China: (1) emphasize principle and precaution; (2) coordinate innovation and regulation; (3) Enhance the importance and involvement of the judicial antitrust function.

## 7 Limitation

The significance of this study is providing suggestions for improving Chinese pharmaceutical antitrust regulation. Nevertheless, some limitations should be noted. First, this study is limited by some undisclosed cases and differences between China and the United States in terms of national conditions, culture, legal terms, case openness and transparency, which may affect the comparability of cases and the depth of micro-comparisons. The future research will be further in-depth through questionnaires, interviews, and other methods. Second, the position of this study is to provide suggestions for the improvement of Chinese pharmaceutical antitrust regulation, focusing on the analysis of the U.S. system for China. However, due to the limitations of the article length and the author’s ability, it fails to systematically deconstruct the inherent contradictions of the U.S.’s pharmaceutical antitrust system. The subsequent research needs to add the bidirectional critical analysis. Third, the case scope of this study is up to 2024, and the development of antitrust regulation after 2024 has not been included. Hence, the conclusions will need to be adopted critically with policy updates.

## Abbreviations

GDP, gross domestic product; FTC, Federal Trade Commission; DOJ, Department of Justice; API, active pharmaceutical ingredient; SAMR, State Administration for Market Regulation; R&D, Research and Development; CNY, Chinese Yuan.
